# Electrochemical Strategies for Lignin Valorization: Advancing Biomass Utilization

**DOI:** 10.3390/molecules31122109

**Published:** 2026-06-15

**Authors:** Filemon Jalu Nusantara Putra, Aliyah Aliyah, Prihardi Kahar, Chiaki Ogino

**Affiliations:** Department of Chemical Science and Engineering, Graduate School of Engineering, Kobe University, 1-1 Rokkodaicho, Nada-Ku, Kobe 657-8501, Japan

**Keywords:** electrocatalytic lignin conversion, biorefinery technologies, biomass valorization, electrochemistry, waste-to-value processes, lignin

## Abstract

Lignin is the most abundant renewable source of aromatic carbon, and yet it remains a mostly underutilized byproduct of the biorefinery and paper industries. Factors such as complexity and a heterogeneous structure make lignin recalcitrant to conventional valorization, the utility of which often requires harsh conditions and expensive catalysts. Electrochemical conversion has emerged as a highly promising, sustainable alternative due to the use of electricity produced by renewable sources to drive depolymerization under mild, ambient conditions. This review summarizes recent progress in this field and provides a comprehensive overview of the primary electrochemical pathways used to promote the valorization of lignin. Herein, we critically examine oxidative strategies that include both direct electrooxidation at the anode surface and indirect oxidation using redox mediators, and provide details of the key challenges of electrode deactivation and product overoxidation. We then discuss reductive strategies with a focus on electrocatalytic hydrogenolysis for C-O bond cleavage. Furthermore, we explore advanced integrated systems that combine electrochemistry with microbial, enzymatic, and photochemical processes to enhance selectivity and efficiency. Finally, this review addresses persistent challenges and offers future perspectives and suggests opportunities with an emphasis on the critical need for innovations in electrocatalyst design, green electrolytes, and integrated reactor engineering to unlock the full potential of lignin as a renewable feedstock for a circular carbon economy.

## 1. Introduction

The demand for sustainable materials and renewable energy has intensified global efforts to develop biorefinery systems that could efficiently convert lignocellulosic biomass into high-value products [1]. Among the three major components of biomass, cellulose, hemicellulose, and lignin, the most abundant renewable source of aromatic carbon in nature is lignin. Due to its structural heterogeneity, complex bonding patterns, and recalcitrant nature, however, lignin is often underutilized and frequently is burned for low-grade heat or energy recovery in the pulp and paper industries. This limited utilization contrasts sharply with lignin’s enormous potential as a feedstock for bio-based chemicals, polymers, and fuels [2]. As a heterogeneous polymer with a variety of recalcitrant C-O and C-C linkages, its deconstruction is challenging. Traditional thermochemical and catalytic approaches for lignin depolymerization, such as pyrolysis, hydrogenolysis, and oxidative cleavage, typically require harsh conditions, expensive catalysts, or non-renewable oxidants/reductants, all of which compromise process sustainability [3]. In recent years, electrochemical conversion technologies have emerged as promising alternatives to address these challenges. By employing electricity potentially derived from renewable sources as the driving force, electrochemical systems use mild conditions to precisely control oxidation and reduction potentials to enable selective bond cleavage of lignin’s β–O–4 linkages and other ether bonds [4,5].

Electrochemical strategies not only enhance reaction selectivity and energy efficiency but also provide a direct link between biomass valorization and the integration of renewable electricity, which contributes to a broader vision of a circular carbon economy. Moreover, the use of electrocatalysts, photoelectrochemical systems, and paired electrolysis allows for the simultaneous generation of value-added aromatic compounds from lignin and green hydrogen at the cathode, which will improve the overall economics of processing. Despite rapid advancements, the electrocatalytic valorization of lignin still faces challenges such as the complexity of lignin feedstocks, low product selectivity, and a limited understanding of reaction mechanisms at the electrode interface. Addressing these barriers requires a combination of materials innovation, mechanistic studies, and process integration within the biorefinery framework. This review summarizes recent progress across this multidisciplinary field. Herein, readers will find a detailed analysis of oxidative and reductive pathways, an exploration into the frontiers of integrated hybrid systems, and critical perspectives on the key challenges and future opportunities in catalyst design, electrolyte engineering, and reactor configurations that are required for a transition to electrochemical lignin valorization in both the laboratory and industry.

## 2. Biomass and Lignin Structure

The agricultural biomass, also known as lignocellulosic biomass, is mainly composed of hemicellulose, cellulose, and lignin with a complex and recalcitrant structure [6]. Among these major components, lignin plays an important role in determining the resistance of biomass to pretreatment, enzymatic hydrolysis, and chemical conversion [7]. Hence, understanding the structural and physicochemical characteristics of lignin is fundamental to developing better and more effective biomass conversion technologies.

### 2.1. Lignin Physicochemical Characteristics and Their Effect on Biomass Conversion

**Figure 1:**
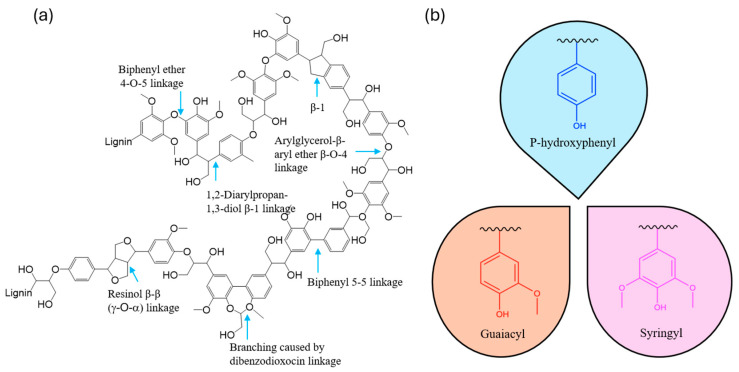
The structure of lignin with the typical linkage and position of a lignin polymer (**a**). The relative structure of the three lignin units (**b**) [9,14].

Unlike hemicellulose, cellulose, or other biopolymers constructed with structures of regularly repeating units, lignin has no common repeating pattern, and subunits such as monolignol are randomly linked [8]. Lignin is a heterogeneous aromatic polymer formed via the radical polymerization of the major monolignols, *p*-coumaryl, coniferyl, and sinapyl alcohols, that correlate to *p*-hydroxyphenyl (H), guaiacyl (G), and syringyl (S) units, respectively (Figure 1). The ratio of these monomers varies among different types of biomass or plants (e.g., hardwoods are rich in S units, while softwoods are G units dominant) [9,10], which influences the lignin molecular weight, cross-linking degree, chemical reactivity, hydrophobicity, glass transition temperature (Tg), and functional group composition. These characteristics directly affect biomass deconstruction efficiency. These monolignols are polymerized mostly through β–5, β–O–4, β–β, and β–1 linkages at the position of the side chain to form linear lignin (Figure 1a). Simultaneously, however, linear lignin is cross-linked via 4–O–5 and 5–5 to form a 3-dimensional structure. Among other linkages, β–O–4 is known to be present in approximately 43–65% of lignin. High lignin content and condensed linkages such as β–5 tend to limit enzyme accessibility and inhibit cellulase activity through non-productive adsorption [11,12,13]. Furthermore, lignin-derived phenolic compounds generated during pretreatment can affect the inactivation of enzymes or microbes, which leads to reduced conversion yields. Therefore, selective pretreatment, which includes electrochemical delignification, is crucial for enhancing biomass utilization and improving the production efficiency of both biofuel and biochemicals.

Effective electrochemical conversion of lignin relies heavily on understanding its specific structural features rather than its general macromolecular form. The abundance of β–O–4 linkages is particularly critical, as these ether bonds are the primary cleavage sites for targeted depolymerization [15]. Meanwhile, lignin’s inherent structural heterogeneity and diverse reactive functional groups dictate its varied redox potentials and electron-transfer mechanisms, which often complicates product selectivity. Furthermore, the recalcitrant nature of lignin introduces severe solubility challenges that restrict mass transfer at the electrode surface [16]. By highlighting these specific factors, it becomes evident that overcoming solubility barriers and targeting key structural bonds are the primary drivers in optimizing current electrochemical conversion strategies.

### 2.2. Lignin-Carbohydrate Complex as a Limiting Electrochemical Conversion Factor

Inside biomass, specifically plant cell walls, lignin and carbohydrates are covalently bonded to form lignin-carbohydrate complexes (LCCs), which is a major contributor to the recalcitrance of biomass when attempts are made to utilize the carbohydrates. LCCs are composed of the ester and ether linkages between the lignin phenolic groups and the carbohydrate hydroxyl or carbonyl groups. Among the linkages, the most common bonds are benzyl-ether (C–O–C), benzyl-ester (C–O–CO–), and phenyl-glycosidic (C–O–C) [17,18,19]. These linkages anchor lignin to hemicellulose and cellulose, which creates physical and chemical barriers that limit enzyme accessibility. Hence, disrupting LCC formation is an important factor in pretreatment methods. Both spectroscopic and chromatographic analysis (NMR, GC/MS, LC/MS, GC-FID, DFRC) continue to reveal the structural diversity of LCCs to determine pretreatment strategies and engineer lignin structures for improved biorefinery applications [20,21]. The presence of residual lignin-carbohydrate complexes (LCCs) significantly impedes electrochemical conversion. The bulky carbohydrate moieties not only introduce a level of steric hindrance that restricts lignin access to the electrode surface, LCCs also significantly limit the overall solubility of the macromolecules in standard electrolytes, which necessitates the development of tailored solvent systems in order to facilitate efficient electron transfer.

## 3. Oxidative Electrochemical Pathways

**Figure 2:**
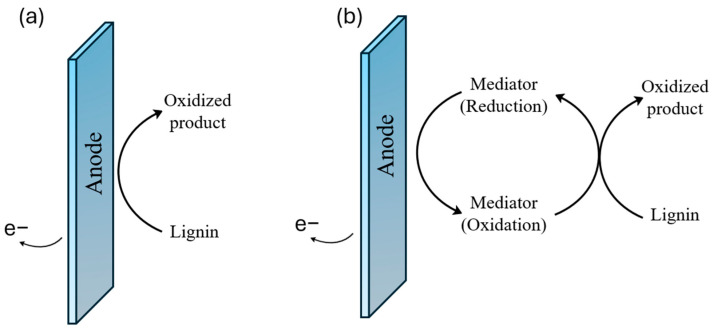
The lignin conversion via oxidative direct electrochemical (**a**) and indirect electrochemical (**b**) reactions [30].

Extensive study has resulted in electrochemical oxidation (electrooxidation) at the anode being the general approach for lignin conversion. The process utilizes anodic potentials to depolymerize the lignin polymer mainly by targeting α-carbonylation and unit linkages C–O and C–C to produce low molecular weight (LMW) aromatic chemicals [22,23]. There are two main electrooxidation mechanisms: the first is where lignin reacts directly on the electrode (direct reaction) (Figure 2a); and the second is where a redox mediator shuttles electrons between the electrode and lignin (indirect reaction) (Figure 2b). For direct electrooxidation, the electrode typically employs a heterogeneous catalyst such as RuO_2_, PbO_2_, or Ni [24,25,26]. These catalysts can also be immobilized on the electrode surface. Therefore, depolymerization with electrolysis occurs on the surface of the electrode. Hence, the electrolyte’s electrochemical stability, lignin solubility, and electron or proton conduction become major obstacles in this process. Unlike direct electrooxidation, the indirect electrooxidation of lignin typically employs homogeneous catalysts such as ferric chloride, *N*-hydroxyphthalimide, or polyoxometalates [16,27,28]. These catalysts are able act as both a proton and/or an electron as well as an oxidant. When the catalyst oxidizes lignin to produce or convert to another chemical product, it is reduced under an applied voltage, at which point it is regenerated at the anode. In order to react with the homogenous catalyst at the anode, lignin could be included in the electrolyte in the form of a pulp.

### 3.1. Lignin Direct Oxidations

Specifically in direct electrooxidation, either the lignin polymer or the model compounds go through the electron transfer directly at the surface of the anode. A heterogeneous catalytic reaction occurs on the anode and depends on the surface structure and the potential applied to it [29]. This section addresses various important aspects related to the direct electrooxidation of lignin, which includes lignin functionalization, depolymerization reactions and mechanisms, anode electrocatalysts, electrolytes for lignin dissolution, and the challenge of direct electrooxidation.

#### 3.1.1. Reactions and Mechanisms

When lignin is oxidized via direct electrooxidation, two main competing outcomes are expected: functionalization and depolymerization. However, side reactions that are involved in direct electrooxidation also occur. Functionalization often involves the selective oxidation of the α-hydroxyl group in the β-O-4 linkage to an α-carbonyl [11,23]. This step is highly desirable as it weakens the C(β)–O bond to subsequently facilitate cleavage, which also prevents any undesirable condensation reactions. The second outcome is depolymerization, which involves the direct cleavage of mainly C–O or C–C (Cα–Cβ) bonds. This reaction leads to the formation of monomeric products. However, these valuable monomer products are typically phenolic compounds that usually are oxidized at the anode, which can lead to undesirable side reactions. The most notable of these reactions is radical coupling, which possibly re-polymerizes the products into an insulating film on the electrode surface that tends to ruin the process [31,32]. A significant advantage of this direct electrooxidation process is the potential for paired electrolysis. As lignin is oxidized at the anode, protons in the electrolytes (or from water) are reduced at the cathode to make a relatively high-purity hydrogen gas (H_2_). The lignin-assisted water electrolysis significantly lowers the thermodynamic voltage required to create H_2_ from around 1.23 V to as low as 0.21 V. This stops the competing oxygen evolution reaction and makes the process more energy efficient and cost-effective overall [33,34,35].

#### 3.1.2. Anode Materials

In the direct oxidative electrochemical processing of lignin, the oxidation reactions primarily occur on the anode surface. Therefore, selecting suitable anode materials is important for maximizing lignin conversion while minimizing undesired side reactions [29]. Ideal anode materials should exhibit high electrical conductivity, good electrochemical stability, and strong resistance to corrosion under oxidative conditions. Various classes of anode materials have been investigated, and these include transition metals and metal alloys such as nickel (Ni), cobalt (Co) [36,37,38], iron (Fe) [37,39], and titanium (Ti) [40], as well as noble metals such as platinum (Pt). Metal oxides such as PbO_2_, SnO_2_, NiCo, NiOOH, and mixed metal oxide (MMO) anodes, have also been widely used for lignin depolymerization [41,42,43].

Among these materials, Ni-based electrodes are widely used because of their low cost, stability, and favorable catalytic activity for lignin depolymerization. Their catalytic performance is highly dependent on the electrode morphology, which affects the electrochemically active surface area, mass transfer, and reactant distribution near the electrode surface. A previous study reported several Ni electrode configurations such as that of a wire, foam, a plate, and felt. Among them, Ni foam exhibited the highest surface area and achieved superior lignin depolymerization compared with the others [44]. Another promising metal for use in direct lignin depolymerization is cobalt (Co). In particular, Co electrodes demonstrate selective conversion of lignin into aromatic aldehyde monomers such as vanillin, and have shown selectivity as high as 58.7% [43,45].

Mixed materials oxide (MMO), also known as dimensionally stable anodes (DSAs), has attracted considerable attention because of its excellent electrical conductivity and resistance to corrosion [46]. A major distinction among MMO electrodes lies in their classification as either active or inactive anodes, depending on their interaction with hydroxyl radicals (·OH). Inactive anodes such as Ti/Sb–SnO_2_ weakly absorb ·OH radicals, which results in highly reactive free radicals that promote non-selective oxidation and deep mineralization of lignin into either low-molecular-weight compounds or CO_2_. By contrast, active anodes such as Ti/PbO_2_ and IrO_2_ interact strongly with ·OH radicals to generate surface-bound oxide species that favor more controlled and selective pathways [47,48,49]. Consequently, active anodes are generally more suitable for selective lignin depolymerization and aromatic monomer production, whereas inactive anodes are more commonly associated with complete oxidation processes. Furthermore, electrode modification such as incorporating PbO_2_ onto multi-walled carbon nanotubes (MWCNTs) is known to improve both catalytic activity and electrochemical stability [50].

Carbon-based electrodes such as graphite, glassy carbon, and boron-doped diamond (BDD) are also attractive because of their wide potential windows, unique surface chemistry, and high resistance to surface fouling [24]. Depending on the applied potential and radical generation behavior, these electrodes could either support selective oxidation or promote extensive mineralization. Therefore, the selection of anode materials should ultimately be tailored according to the targeted product distribution and oxidation pathway.

#### 3.1.3. Key Challenges

Despite its potential, direct oxidation faces several major challenges. The first is anode deactivation, which presents a significant barrier. In such a development, the polymerization of phenolic compounds on the anode forms an insulating layer that blocks active sites and leads to a rapid decrease in both the reaction rate and current efficiency [51,52]. Lignin solubility in the system also becomes a challenge. While alkaline solutions (NaOH) are electrolytes, they tend to limit the anodic potential window due to the onset of water splitting. Ionic liquids (ILs) offer an alternative to high lignin solubility and a wider electrochemical window. However, ILs are significantly more expensive than alkaline solutions that are considered unsuitable for commercial-scale systems [53,54,55]. Another challenge presented by this approach is the presence of products from overoxidation processes. The desired low molecular weight monomers are often more easily oxidized than the original lignin polymer, and this leads to their degradation into smaller molecules, acids, or CO_2_, which drastically affects the product yield [16]. Therefore, to avoid this condition, several co-product removal strategies have been developed to rapidly extract the desired low-molecular-weight monomers from the oxidative environment. These include the use of continuous ultrafiltration membrane reactors [56], liquid–liquid extraction with immiscible solvents [57], and direct product adsorption onto anion-exchange resins [58]—although the physical setups are still operated at lab scale. However, these strategies are explicitly designed as proofs-of-concept for continuous electrolysis and offer strong evidence for scalability.

### 3.2. Lignin Indirect Oxidation

Indirect electrooxidation appeared to overcome the primary challenges found in direct electrooxidation systems. In this system, either the redox mediator or the electron shuttle is oxidized at the anode. This activated mediator then diffuses into the bulk solution, where it chemically and selectively oxidizes the lignin substrate. The current-reduced mediator diffuses back to the anode to be regenerated and completes the catalytic cycle [4,59]. The main advantage of this systematic approach is the spatial separation of the electrode from the bulk lignin polymer. This completely circumvents the issue of electrode fouling as the lignin and its polymerization-prone products will never touch the anode surface, which is expected to allow for a sustained and longer operation. Furthermore, the mediator itself could be chosen to target specific chemical bonds that offer a much higher degree of selectivity compared with that of direct oxidation [60,61].

#### Mediator Systems

In a few years, a variety of redox mediators have been successfully employed specifically for lignin valorization [4]. Nitroxyl radicals or amonoxyl mediators such as TEMPO (2,2,6,6-tetramethylpiperidine-N-oxyl) and N-hydroxyphthalimide (NHPI) are considered highly effective since they are particularly adept at the selective oxidation of the α-hydroxyl group in non-phenolic β-O-4 linkages to the α-carbonyl. This pre-oxidation step is crucial for facilitating the subsequent depolymerization. Studies have shown that the TEMPO-mediated oxidation of real lignin extracts produces a polyelectrolyte material that, upon subsequent depolymerization, produces high yields of monomers [62,63,64].

Also, there is a one-pot system that combines NHPI-mediated electrocatalysis with photocatalytic C-O bond cleavage. This approach has also been used for the controlled oxidation and depolymerization of lignin by sequentially combining electrocatalysis and photoredox catalysis [1]. The method utilizes NHPI as a hydrogen atom transfer mediator in an electrocatalytic step to selectively oxidize benzylic alcohols within β-O-4 linkages into ketones. This is immediately followed by a photocatalytic C-O bond cleavage step using an iridium-based catalyst and a sacrificial electron donor, which enables the efficient fragmentation of both lignin model dimers and native lignin without the need for intermediate isolation. This integrated redox strategy provides a selective and operationally simple route for upgrading recalcitrant biomass into aromatic commodity chemicals while avoiding the harsh temperatures and chemical oxidants typically required for lignin degradation [1,65]. However, polymetalates (POMs) could also be used. POMS are multi-metal oxyanions that are highly efficient electron-proton carriers. In an indirect electrooxidation system, the POM chemically oxidizes lignin at temperatures approaching 100 °C, and reduced POMs are regenerated at the anode in a very low potential. This process simultaneously generates H_2_ at the cathode, which acts as a chemical-electric conversion system with high efficiency [66].

The Fe^3+^/Fe^2+^ redox couple has been used to oxidize either kraft or alkaline lignin, with Fe^2+^ generated at the anode. This system produces good lignin, with around 16% of small aromatic molecules [4,67]. Another material is halides. Halide ions, particularly iodide, mediate the selective cleavage of Cβ–O bonds in lignin model compounds, which offers a different pathway for depolymerization [24]. Such a variety of redox mediators mandates selection of both an appropriate mediator and reaction conditions. Indirect electrooxidation provides a robust and highly tunable platform for converting lignin into specific and value-added aromatic chemicals.

## 4. Reductive Electrochemical Strategies

**Figure 3:**
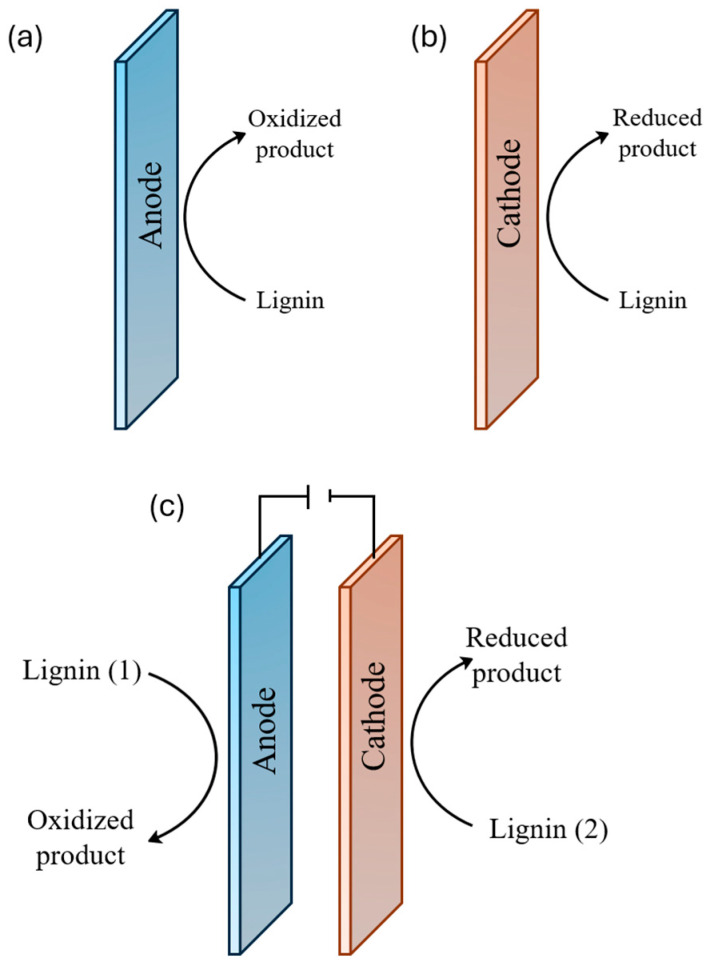
Lignin reaction under oxidative electrochemical (**a**), reductive electrochemical (**b**), and hybrid oxidative-reductive electrochemical (**c**) systems [4].

In contrast to oxidative pathways, electrochemical reduction (electroreduction) at the cathode is less common than electrooxidation (Figure 3). However, it continues to be a highly valuable strategy for lignin conversion. While the oxidation system tends to increase the oxygen content of products, the reduction system increases the hydrogen and carbon content, which is highly desirable for producing biofuels with a higher combustion value [68,69,70]. The primary reductive approach is electrocatalytic hydrogenolysis (ECH), which provides an environmentally mild alternative to traditional thermal catalytic hydrotreating processes that require temperatures ranging from 180 to 270 °C and pressures that can reach 77 bar with an external supply of hydrogen (H_2_) gas [71,72]. In ECH, the chemisorbed hydrogen is generated in situ specifically on the cathode surface by the electrochemical reduction in either the protons or water (H_2_O). Then, this highly reactive atomic hydrogen is used to directly react with the adsorbed lignin substrate available in the system [69,73,74].

### 4.1. Reaction Pathways

The ECH of lignin involves two main competing reaction pathways. First is hydrogenolysis, which involves cleavage of the C-O single bonds (either β-O-4 or 4-O-5) of ether linkages, to produce monomeric phenols. The second one is hydrogenation. This is a subsequent reaction where the aromatic rings of the newly formed phenol compounds are saturated by hydrogen at the cathode. Hydrogenation pathways are known to be dominant in converting the valuable aromatic monomers into either cyclohexanols or other cycloalkyl ethers. One major challenge in ECH is tuning the catalyst and conditions to favor hydrogenolysis over hydrogeneration. Both the distribution of the products and the production yield are controlled by varying the cathode material, the current density, the electrolytes, and the temperature [75,76,77,78].

### 4.2. Cathode Materials

The cathode material is the most critical factor in determining both the ECH pathway and the product selectivity. The specific product distribution in ECH is fundamentally dictated by the catalyst class and its distinct hydrogen adsorption mechanisms. Conversely, non-noble transition metals such as Nickel and Copper (Cu) possess moderate amounts of atomic hydrogen binding energy. This distinct mechanistic feature allows these catalysts to selectively promote the hydrogenolysis of ether linkages (yielding phenolic compounds) while resisting a complete saturation of the aromatic ring. One of the most widely studied materials is Raney nickel (Ra-Ni). This material shows significant levels of activity in the hydrogenolysis of phenolic β-O-4 lignin model compounds, with effective cleavage of the ether linkage to produce phenolic compounds and their hydrogenated derivatives [69,79,80,81].

Noble metals typically exhibit high levels of affinity for both atomic hydrogen and the aromatic ring. This property drives a rapid and relatively indiscriminate hydrogenation process, which often leads to completely saturated products such as cyclohexanol. Ruthenium supported on activated carbon cloth (Ru/ACC) is known to be effective for cleaving the 4-O-5 model compounds. However, this catalyst strongly favors hydrogenation and yields cyclohexanol as the primary product [77]. Furthermore, cathode materials play a vital role in paired electrolysis systems where mixed oxidative/reductive behaviors occur. For instance, in the electrochemical degradation of raw biomass to yield oxidative products such as vanillin and syringaldehyde, the anodic reactions drive depolymerization. In such systems, copper (Cu) cathodes have proven to be highly effective. The Cu cathode is advantageous because it provides the necessary redox balance while limiting excessive hydrogenation, thereby preventing the over-reduction in the valuable aldehyde products into their corresponding alcohols [82,83]. Another system that uses sodium borohydride (NaBH_4_) is known to have achieved reductive cleavage of aryl ethers at room temperature. Furthermore, three-dimensional electrode reactors made from copper-mesh cathodes have been developed to improve catalytic performance for the hydrocracking of lignin [84]. Although ECH is a promising strategy for lignin depolymerization under mild and ambient-pressure conditions, further catalyst development is required to precisely control the selectivity between C-O bond cleavage and aromatic saturation.

Another method combines hybrid oxidation and reductive electrochemical systems for lignin conversion (Figure 3c). These systems utilize a paired electrolysis configuration to maximize energy efficiency and product value by simultaneously performing useful chemical transformations at both the anode and the cathode [85,86]. In these dual-functional setups, the anodic chamber typically facilitates the oxidative depolymerization of lignin, either through direct electron transfer or mediated pathways, to yield aromatic monomers such as vanillin and syringaldehyde [87]. Concurrently, the cathode drives a reductive process, most commonly the hydrogen evolution reaction (HER), either for green fuel production or the generation of reactive species such as H_2_O_2_ that further assist in the oxidation process [4,88]. This integrated approach ensures that every electron transferred through the external circuit contributes to a value-added product, thereby optimizing the atom economy and economic viability of the biorefinery process.

## 5. Integrated System of Electrochemical Depolymerization

Integration is used to overcome the limitations of a single standalone system for either electrochemical oxidation or a reduction in lignin. Current research is increasingly focused on integrated systems that combine electrochemical methods with other approaches—biological methods using either microorganisms or enzymes combined with photochemical processes [26,89]. The criteria for inclusion in these integrated systems are based on the commonality of oxidative interfacial electron-transfer mechanisms. Here, we include not only systems aimed at large-scale monomer production but also proof-of-concept platforms in order to provide critical insight into the oxidative activation of recalcitrant lignin bonds at the electrode/electrolyte interface. The aims of these hybrid approaches are to improve selectivity, enhance conversion rates, and utilize energy sources such as solar to increase efficiency (Table 1).

### 5.1. Microbial Electrochemical Conversion of Lignin

Microbial electrochemical conversion uses three types of systems: microbial fuel cells (MFCs), microbial electrolysis cells (MECs), and microbial electro-fenton (MEF). These three systems offer unique pathways for lignin valorization wherein microorganisms catalyze the degradation of complex organic molecules [90,91]. These three technologies all belong to the family of microbial electrochemical technologies (METs) and all utilize electroactive microorganisms at the anode, but they differ fundamentally in both their thermodynamics and in the chemical work they perform.

#### 5.1.1. Microbial Fuel Cells (MFCs)

MFCs make up a bioelectrochemical system that directly converts the chemical energy of organic substrates into electricity. A solution of microbes form a biofilm on an anode, and the organic substrates are oxidized to release protons into the solution and electrons to the anode. The electrons then flow through an external load to the cathode, where they reduce an electron acceptor [92]. This is a spontaneous process that releases energy (Δ*G* < 0), which represents the potential difference between a low-potential anode and a high-potential anode to drive the flow of electrons. The cathode reaction in this system is usually the oxygen reduction reaction (ORR), because oxygen is abundant and has a high redox potential [93].

O_2_ + 4H^+^ + 4e^−^ ⟶ 2H_2_O (1)

**Figure 4:**
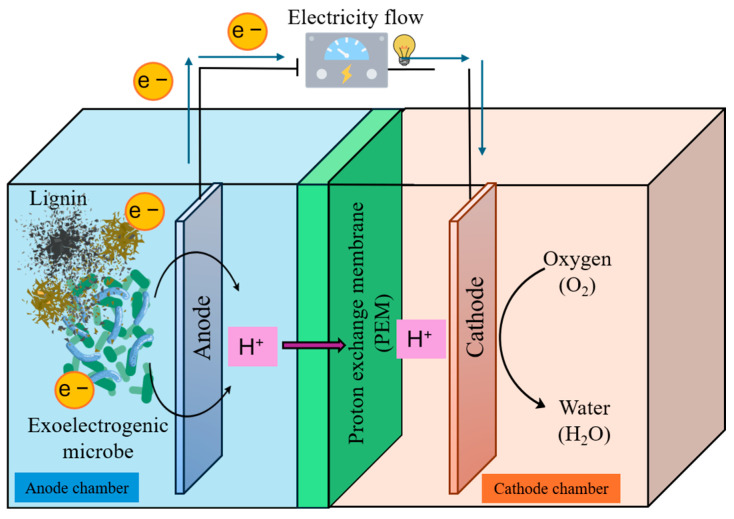
Relative schematic of a microbial fuel cell for spontaneous electricity generation from lignin [94].

Hence, MFCs produce a net surplus of electricity while simultaneously treating wastewater or processing biomass, which is the beneficial object of this system (Figure 4).

Sharma et al. [95] reported that they successfully developed a self-sustainable, dual-chambered MFC designed for the H_2_O_2_-mediated oxidative depolymerization of lignin without the need for an external power source. In this electrochemical system, the exoelectrogenic bacterium *Shewanella putrefaciens* is inoculated into the anodic chamber. The anolyte consists of a glucose-yeast extract medium that serves as the electrolyte, and carbon fiber cloth is used as the anode material. The cathodic chamber uses a stainless-steel mesh cathode that contains alkali-extracted wheat-straw lignin in 0.2 mol/L of a NaCl solution (pH 4.5). The system has exhibited a maximum power density of 0.58 μW cm^−2^ over 2 days of operation. The cathodic generation of H_2_O_2_ effectively cleaves the β-O-4 aryl ether linkages in the lignin polymer. This degradation process produces vanillin as the principal value-added aromatic compound. Sukri et al. [96] explored a membraneless, single-chamber microbial zinc/air cell configuration for sustained electricity generation from an empty fruit bunch (EFB). This system uses the white-rot fungus *Phanerochaete chrysosporium* to biodegrade EFB, which serves as the lignin source. The electrochemical setup consists of a high-purity zinc foil anode paired with a commercial air electrode cathode that are submerged in a simple, unbuffered potato dextrose broth (PDB) solvent. Rather than extracting specific chemical monomers, the main functional product of this system is the continuous in situ secretion of the laccase enzyme that is the result of the fungal decomposition of lignin. This system features sustained enzymatic activity that facilitates the benign reduction in molecular oxygen to water at the cathode to yield an impressive level of electrocatalytic performance wherein the cell is known to maintain a continuous 1 mA discharge current for 44 days to ultimately achieve a total discharge capacity of 1056 mAh. Together, these studies illustrate how tailoring the electrode configuration, microbial consortium, and electrolyte environment allows MFCs to flexibly adapt to either a level of fine biochemical synthesis or to robust bioenergy recovery from recalcitrant lignin streams.

#### 5.1.2. Microbial Electrolysis Cells (MECs)

MECs make up an electrolyte system that produces chemical products from organic matter—most commonly hydrogen gas (H_2_). The mechanism is similar to that of MFCs. The microorganism oxidizes organics at the anode. However, the reduction in protons to hydrogen at the cathode is a non-spontaneous process that requires energy input (Δ*G* > 0) with the potential provided by the bacteria alone [97,98]. Therefore, the MECs require the addition of an external power source to the system or circuit. The cathode reaction in this system is the reduction in protons by the hydrogen evolution reaction (HER).

2H^+^ + 2e^−^ ⟶ H_2(g)_ (2)

Because the microorganisms at the anode significantly reduce the required energy input, bio-hydrogen production is much more efficient than that from standard water electrolysis. In a recent study, a pilot-scale 13 L multi-stack dual MEC system was developed to valorize complex biomass. This system utilizes a graphite felt anode coupled with a titanium (Ti) mesh cathode. Serving as a sustainable source of both lignin and carbohydrate, the system has been used to process a mixture of fruit waste (MFW) consisting of apples, bananas, pineapples, and orange peels pretreated with citric acid. In that procedure, the solvent/electrolyte environment consisted of phosphate-buffered saline (PBS) containing the pretreated MFW extract. Strains from the *Geobacter* and *Comamonas* genera were used with an anaerobic system. MEC operation with 25% MFW achieved a current density that reached 0.71 A/m^2^ (16.36 A/m^3^) with a chemical oxygen demand (COD) removal efficiency of 77%. The main value-added product of this system was hydrogen gas, which reached a hydrogen production rate (titer) of 0.02 L/L·d [99].

Other researchers have used the MEC system as a strategy to enhance lignocellulose conversion into humic substances. In one such study, a 50 L reactor was utilized to process a 1:1 mixture of mushroom residue and pig manure. This electro system used graphite electrodes under 5 V of direct current. The moisture content was maintained at 60% using water as the electrolyte medium. The fungus *Aspergillus oryzae* was used under electric field assistance, with a propagation rate that was increased by 29.9%. The synergy between the electric field and the fungal inoculum increased laccase catalytic activity by 74.9%, which contributed to a final lignocellulose degradation rate of 62.2%. The main value-added product was composed of humic substances that reached a significant titer of 155.7 g/kg, which was a 20.2% increase compared with that of conventional composting. In this case, the MEC system proved to be an efficient path for converting agrowaste into high-quality soil conditioners [100].

#### 5.1.3. Microbial Electro-Fenton (MEF)

MEF is a hybrid system designed for the advanced oxidation of highly recalcitrant or toxic compounds, such as complex lignin polymers or industrial dyes [101]. The MEF system operates by generating an in situ chemical oxidant. The system is designed to produce hydrogen peroxide (H_2_O_2_) at the cathode through a two-electron reduction of oxygen [102]. The MEF reaction occurs when iron catalysts (Fe^2+^) are present in the catholyte and they react with the electro-generated H_2_O_2_ to produce hydroxyl radicals ·OH. These radicals are among the strongest known oxidants and can non-selectively break down almost any organic bond [103].

O_2_ + 2H^+^ + 2e^−^ ⟶ H_2_O_2_ (3)

Fe^2+^ + H_2_O_2_ ⟶ Fe^3+^ + ·OH + OH^−^ (4)

**Figure 5:**
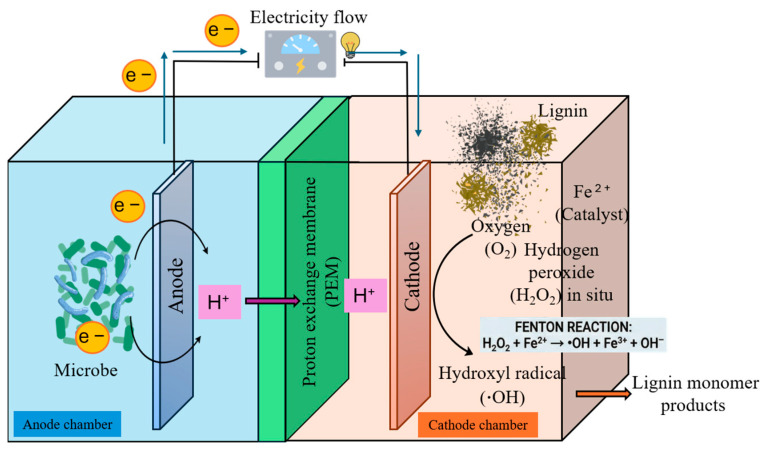
Relative schematic of a microbial electro-fenton (MEF) system for lignin [104].

This system provides deep mineralization of complex waste that microorganisms cannot digest on their own, and it breaks up the polymer so it can be further processed (Figure 5).

The MEF system has demonstrated significant efficacy in depolymerizing lignin extracted from agricultural residue such as rice straw. In a study by Chang and Gupta [90], a microbial peroxide-producing cell (MPPC) was utilized to achieve in situ depolymerization while simultaneously treating wastewater. This oxidative electro system generated electrochemical voltage yields of 0.171 ± 0.05 to 0.497 ± 0.2 V, which successfully produced H_2_O_2_ concentrations ranging from 9 to 34 mM. The system utilized a mixed electroactive microbial consortium to drive anodic oxidation, and achieved biological and chemical oxygen demand (BOD/COD) removal rates of 60 to 85%. The resultant H_2_O_2_ drove the cleavage of the lignin structure, primarily producing value-added aromatic compounds such as carboxylic acid derivatives, benzopyran, and hexanoic acid.

In a similar manner, Mukhopadhyay et al. [90] found that this self-sustaining electrochemical cell requires no external energy input to achieve a maximum open-circuit voltage of 1.143 V and current and power densities of 14 mA/cm^2^ and 13 mW/cm^2^, respectively. The electrocatalytic performance facilitated the production of 26 mM of H_2_O_2_, which was used in the degradation of critical β-β and -O-4 linkages. The primary valuable products identified included vanillin, p-coumaric acid, and ferulic acid, which confirms the potential of MPPCs for controlled lignin depolymerization.

The specific production of high-value bioflavors such as vanillin has also been achieved through MEC-assisted depolymerization of Kraft lignin. Chandrawanshi et al. [105] introduced an integrated approach combining MEC-based oxidation with ultrafiltration and liquid–liquid extraction for efficient product recovery. The system produced a power output of 224 ± 0.08 mV and 29.20 ± 0.39 mM of H_2_O_2_ that resulted in an 89% reduction in COD. This process achieved a bioconversion efficiency of 16.9%, and yielded vanillin with 99% purity and a significant titer of 146 mg/g of Kraft lignin.

### 5.2. Electroenzymatic Conversion of Lignin

**Figure 6:**
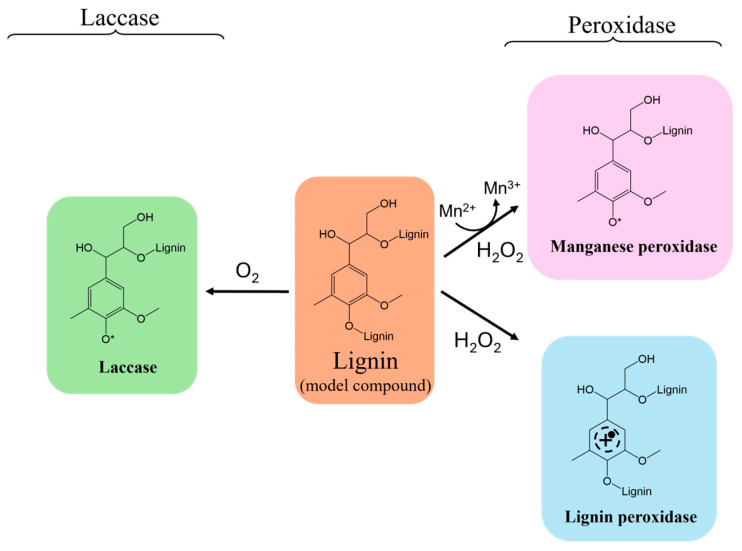
Enzymes used in the integrated electroenzymatic conversion of lignin [9]. The asterisk (*) symbol on the oxygen atom denotes an unpaired electron, representing a free radical (specifically, a phenoxy radical intermediate) generated during the laccase-catalyzed oxidation of lignin. As well as in the manganese peroxidase.

Electroenzymatic lignin conversion is an advanced hybrid approach that integrates the catalytic specificity of redox enzymes with the electrochemical cell [106]. In this oxidation system approach, ligninolytic enzymes such as laccase, manganese peroxidases, and the versatile peroxidase—or engineered variants (Figure 6)—are immobilized on conductive electrode materials such as graphite felt, glassy carbon, or carbon nanotube (CNT) nanocomposites to facilitate either direct or mediated electron transfer to then catalyze an oxidative cleavage of the aromatic polymer structure of the lignin complex [107]. The electrode serves as an electron donor or acceptor that enables an efficient regeneration of the enzyme’s redox that eliminates the need for large amounts of chemical oxidants. In this process, the enzymes oxidize phenolic and non-phenolic lignin units to generate reactive radicals that lead to the breakdown of key interunit linkages such as the β-O-4 bond. This controlled depolymerization yields valuable aromatic products such as vanillin, syringaldehyde, and other phenolic derivatives. Compared with conventional lignin depolymerization, the electroenzymatic systems offer much milder conditions, provide higher reaction selectivity, and allow improved control of electron transfer processes [23,108]. As a result, this system represents a promising green technology for integrating biocatalysis and electrochemistry.

A recent study demonstrated a significant advancement in this area by engineering a laccase from *Bacillus pumilus* for improved thermostability. In this enzymatic system, site-saturation mutations (A347H and N368L) resulted in activity increases of 2.37 and 2.46-fold, respectively, allowing the enzymes to remain active at temperatures up to 90 °C. The enzymes, when combined with lytic polysaccharide monooxygenase and ascorbic acid, facilitate the depolymerization of alkali lignin without the need for an external mediator and improve depolymerization efficiency by approximately 40% [109]. In another integrated approach, Saikia et al. [110] developed a system that utilizes a co-immobilized dual-enzyme strategy that pairs laccase with versatile peroxidase on magnetic silica microspheres. The magnetic silica allows for the easy recovery and reuse of the biocatalyst, which is a major hurdle in scaling up lignin-to-vanillin conversion. The synergetic integration of these enzymes effectively cleaves complex lignin bonds, such as β-O-4 linkages, to selectively produce vanillin, which provides a robust model for enzyme-mediated electrochemical catholytes.

Researchers have used the laccase-mediator system (LMS) to investigate the enzymatic depolymerization of 3 major industrial lignins: organosolv lignin, kraft lignin, and sodium lignosulfonate. The system used a commercial laccase polyphenoloxidase from the fungus *Trametes versicolor*, combined with mediators TEMPO and 2,2′-azino-bis(3-ethylbenzothiazoline-6-sulfonic acid) (ABTS). The low solubility of typical industrial lignins was overcome via the use of 25% (*v*/*v*) 1,4-dioxane in a 0.1 M acetate buffer (pH 4.6). Electrocatalytic performance, monitored via Gel Permeation Chromatography (GPC) and mass spectrometry, demonstrated that while either laccase alone or mediators alone were ineffective, the LMS-ABTS couple successfully initiated depolymerization. Results indicated a significant shift toward lower molecular weight aromatic compounds into smaller fragments including vanillin, styrenes, and phenolics [111]. LMS involves the immobilization of laccase (EC 1.10.3.2) with a polydopamine film for the detection and analysis of phenolic compounds. The system employed graphite or glassy carbon as the electrode material, which provided an inexpensive and disposable platform for electrochemical sensing. A citrate-phosphate buffer (pH 4.6) served as the electrolyte that was aerated periodically during measurements to maintain a constant oxygen concentration for the enzymatic reaction. While the system is designed primarily for sensing rather than large-scale production, it effectively targets low molecular weight phenols from industrial waste such as chestnut shell extract. The electrocatalytic performance is highlighted by sensitivity that can reach as high as 19.3 mA M^−1^ cm^−2^ for gallic acid, with a low detection limit of 0.29 µM and a linear range of 1–150 µM. Other main products monitored include caffeic acid and rosmarinic acid, which are common markers for the residual phenolic content in treated lignocellulosic waste [112].

An advanced application of a laccase-based electrochemical system is used in the development of a biosensor for the detection of lignin from dietary fiber. This system utilizes a laccase-ionic liquid and a carbon nanotube paste electrode (lac/IL/CNPE). The electrode material consists of a nanocomposite of carbon nanotubes (CNTs), and the ionic liquid 1-butyl-3-methylimidazolium hexafluorophosphate ([BMIM]PF_6_) serves as a binder and conductive enhancer to provide a highly effective surface area. The laccase enzyme (EC 1.10.3.2), sourced from *Trametes versicolor*, is immobilized onto this nanocomposite surface. The electrochemical system operates in a phosphate-buffered saline (PBS) electrolyte solution at an optimized pH of 4.5. Electrocatalytic performance is evaluated through cyclic voltammetry (CV) and differential pulse voltammetry (DPV) and shows sensitivity as high as 0.92367 µA/mg·mL^−1^ with a detection limit of 1.05 mg/mL and a broad linear range of 0–110 mg/mL. This system successfully detects the electrochemical signal generated by the oxidation of lignin fragments to enable precise quantification of lignin content in various dietary fiber samples such as wheat bran, oat bran, and corn bran [113].

### 5.3. Photoelectrochemical Conversion of Lignin

**Figure 7:**
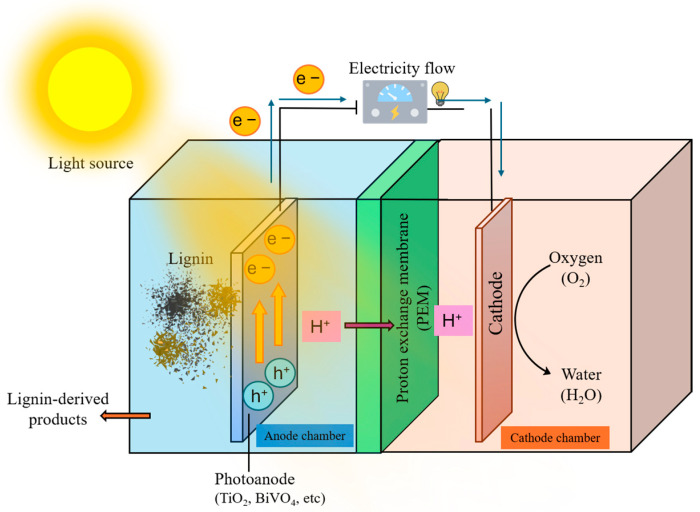
Relative configuration and mechanism of a photoelectrochemical cell for solar-driven lignin conversion [117].

Photochemical systems are often discussed as a solar-thermal electrochemical process that uses light energy to drive or assist in the electrochemical conversion of lignin (Figure 7). This type of approach is highly appealing as it directly utilizes renewable solar energy [114,115]. A recent publication described the use of photoelectrocatalysis to investigate the oxidation of lignin derived from the pulp and paper industry in the form of black liquor. A niobium-doped titanium dioxide (TiO_2_:Nb) photoanode within a photoelectrochemical flow cell system was used. Operating in an alkaline solvent, the system utilized simulated solar radiation and a low constant cell potential of 0.8 V to drive the oxidation process. The electrocatalytic performance showed a charge consumption of 5 C/g of lignin over 7 h of reaction. The primary valuable products were identified as low molecular weight phenolic products—predominantly aromatic acids (26 mg/kg). This research demonstrates a promising transformation of high alkaline industrial waste streams into useful chemical precursors via the use of solar-electrolysis [6].

The potential for extracting aromatic chemical feedstocks from biomass has been further demonstrated through the photoelectrochemical decomposition of lignin model compounds. This system employed a bismuth vanadate (BiVO_4_) photoanode, which was occasionally modified with a V_2_O_5_ layer to improve conversion rates by approximately 10%. The photoelectrochemical cell was operated under simulated sunlight with an applied bias of 2.0 V using the vanadium sites of the electrode as active electrocatalytic centers that eliminated the need for external redox mediators in the solution. The electrocatalytic performance was significant and achieved conversion by as much as 64% of the prototypical lignin model compounds over operation lasting for 20 h. The main products generated through this process were aryl aldehydes and phenol derivatives, and BiVO_4_ was highlighted as an effective platform for solar-driven biomass depolymerization [116].

Another development of an integrated approach was revealed when TiO_2_/lignin-based carbon composite photocatalysts were synthesized to convert kraft lignin into valuable chemicals. The electrode material was fabricated by compositing titanium dioxide with carbon nanostructures derived from kraft lignin thermally treated at 500–1000 °C. In this photo system, UV light was used to initiate the photocatalytic reaction within an aqueous electrolyte. The inclusion of the lignin-based carbon served to suppress the recombination of photogenerated electron-hole pairs, which significantly improved the electrocatalytic performance compared with the use of pure TiO_2_. This system successfully produced a range of aromatic monomers, with vanillin and syringaldehyde as the main valuable products, which proved the efficacy of using lignin-derived materials to catalyze the valorization of the polymer itself [118].

There also is a highly efficient photoelectrochemical system that utilizes a ruthenium dye-sensitized photoanode (DSPEC) that was intended to demonstrate alkene epoxidation while providing a critical framework for the selective oxidation of the complex organic linkages found in lignin. The system utilized a mixed solvent of CH_3_CN/H_2_O with LiBr acting as a redox mediator to facilitate charge separation. Under visible light irradiation, the photo system achieved an excited electron injection efficiency of 95% and a dye regeneration efficiency of 87%. The electrocatalytic performance was characterized by the successful blockage of the charge recombination pathways that lead to high levels of Faradaic efficiency for oxidative transformations. This DSPEC configuration offers a sophisticated blueprint for using light energy to drive selective bond cleavage in biomass-derived substrates and potentially yield high titers of specific oxidized monomers when applied to lignin valorization [119].

**Table 1:** Integrated system of electrochemical lignin depolymerization.

System Type	Electrode Material	Lignin Source	Solvent/Electrolyte	Electro System	Agent (Microbe, Enzyme, Light-Type)	Main Products	Reference
Microbial fuel cell	Carbon fiber anode/graphite cathode	Alkali-extracted wheat straw lignin	Phosphate buffer	Oxidative (H_2_O_2_ generation at cathode)	*Shewanella putrefaciens* biofilm	Vanillin	[95]
	Carbon electrode	Oil palm empty fruit bunch (EFB) lignin	Aqueous medium	Oxidative enzymatic electro-biodegradation	*Phanerochaete chrysosporium*	Phenolic fragments	[96]
Microbial electrolysis cell	Steel electrodes inserted into compost matrix	Lignocellulosic waste	Compost slurry	Oxidative	*Aspergillus oryzae*	Humic substances	[100]
Microbial electro-fenton	Carbon electrodes	Technical lignin	Neutral electrolyte	Oxidative (electro-Fenton)	Mixed electroactive bacteria	Vanillin, ferulic acid, p-coumaric acid	[90]
	Carbon electrodes	Rice straw lignin	Wastewater electrolyte	Oxidative electro-Fenton	Mixed microbial consortium	Aromatic acids, benzopyran derivatives	[90]
Electroenzymatic	Carbon nanotube paste electrode with ionic liquid nanocomposite	Lignin from dietary fiber	Ionic liquid/aqueous electrolyte	Oxidative electro-enzymatic	Laccase	Phenolic lignin fragments	[113]
	Carbon electrode coated with electro-polymerized polydopamine film	Phenolic lignin extracts	Aqueous buffer	Oxidative electro-enzymatic	Laccase	Oxidized phenolic compounds	[112]
	Not electrode-immobilized but adaptable to electroenzymatic systems	Kraft lignin, organosolv lignin, lignosulfonate	1,4-dioxane/water cosolvent	Oxidative	Laccase + mediator (ABTS)	Low-MW aromatics	[111]
	Magnetic silica microsphere support (can be integrated in electrochemical reactors)	Lignin from *Casuarina equisetifolia* biomass	Buffered aqueous system	Oxidative	Laccase + versatile peroxidase	Vanillin	[110]
	Typically immobilized biocatalyst platforms	Alkali lignin	Aqueous alkaline solution	Oxidative	Engineered laccase + LPMO	Aromatic fragments	[109]
Photoelectrochemical	Nb-doped TiO_2_ photoanode	Kraft lignin from black liquor	1 M NaOH alkaline solution	Oxidative photoelectrocatalysis	UVA-LED (365 nm)	Vanillin, syringaldehyde, vanillic acid, syringic acid, acetovanillone	[6]
	BiVO_4_ photoanode (V_2_O_5_ modified)	Lignin β-O-4 model compounds	Aqueous electrolyte	Oxidative PEC	Simulated sunlight	Aromatic aldehydes and phenols	[116]
	TiO_2_–lignin composite photocatalyst electrode	Lignin-derived carbon	Aqueous medium	Oxidative photocatalysis (PEC-compatible)	UVA irradiation	Vanillin and aromatic compounds	[118]
	α-Fe_2_O_3_, ZnO, TaN, SrTiO_3_ photoanodes	Kraft lignin	Alkaline electrolyte	Oxidative PEC	UV (solar light)	Phenolic monomers	[119]

## 6. Molecular Modeling and Computation When Using Lignin Electrochemical Systems

Recent research in the field of electrochemical lignin valorization has highlighted the importance of molecular modeling for understanding the complex physicochemical interactions occurring at the electrode-electrolyte interface. Due to the structural heterogeneity of lignin and the transient nature of reactive intermediates formed during electrocatalytic depolymerization, conventional experimental techniques alone are often insufficient to fully elucidate reaction mechanisms on an atomic scale. In this context, Density Functional Theory (DFT) simulations have emerged as indispensable computational tools for investigating adsorption configurations, electron-transfer behavior, bond dissociation energetics, and catalytic reaction pathways of lignin-derived compounds on various electrode surfaces.

Computational studies mapping the bond dissociation enthalpies (BDEs) across diverse native and modified lignin models reveal a distinct energetic hierarchy [120,121]. Consistently, carbon-oxygen (ether) bonds, particularly the α-O-4 and β-O-4 linkages, have emerged as the most energetically vulnerable sites compared with robust carbon-carbon bonds such as β-5 and 5-5 linkages. Furthermore, these calculations demonstrate how natural variations, such as ortho-methoxy substitutions on arene rings [121] or energetically favored triplet intermediate states during ring-opening [122], induce steric hindrance and electronic delocalization that further lowers the cleavage energy of adjacent bonds. By quantifying these intrinsic thermodynamic properties, molecular modeling establishes a baseline for predicting which specific linkages are most susceptible to targeted electron transfer on an electrode surface.

Beyond mapping native linkages, computational modeling elucidates how structural modifications and solid-catalyst interactions alter lignin’s recalcitrance—mechanisms that closely parallel phenomena in electrocatalytic environments. DFT simulations highlight how the pre-oxidation of aliphatic hydroxyl groups to ketones or aldehydes drastically reduces the BDE of corresponding ether linkages, which suggests that coupled electro-oxidation strategies could significantly lower the overpotential required for subsequent cleavage. Additionally, mechanistic studies of solid catalytic interfaces such as palladium on carbon reveal that naturally occurring pendent groups such as *p*-hydroxybenzoate actively direct reactivity. Through carboxylate-assisted C–H bond activation at the catalyst surface, these groups shift the kinetic preference toward specific ester intermediates and fundamentally bypass traditional cleavage sequences [123]. Understanding these complex reaction pathways on solid surfaces provides a crucial predictive framework for anticipating how lignin molecules will orient, interact, and fragment when subjected to heterogeneous electron-transfer mechanics.

Bridging these DFT-derived thermodynamic and mechanistic insights with electrochemical modeling is essential for advancing targeted lignin valorization. While extensive editions of computational literature have characterized thermal homolysis and traditional heterogeneous catalysis, adapting these workflows to explicitly include applied potentials, solvent-electrode interactions, and double-layer dynamics remains a vital frontier in biorefinery research. By leveraging the fundamental understanding of radical stability and bond fragility established through molecular modeling, a rational design for electrocatalytic systems could be realized. This computation-guided approach enables the precise tuning of electrode materials to selectively cleave specific lignin linkages and thereby maximize the yield of high-value biochemicals while minimizing necessary energy input.

## 7. Challenge, Opportunity, and Future Prospects of Lignin Electrochemical Conversion

Regardless of significant advances, the widespread commercial application of electrochemical lignin conversion remains unrealized. This is due to several persistent challenges that must be addressed, because this will define the key opportunities and future prospects for the field. The main challenge for electrochemical systems is the occurrence of unfavorable side reactions. To achieve practical reaction rates, working potentials are often required. However, these high potentials also promote competing reactions, particularly the electrolysis of water, which generates H_2_ and O_2_ gas. This reaction consumes a significant amount of electrical input and drastically lowers the overall Faradaic efficiency of the lignin conversion process. Lignin is known for its heterogeneous structure, which results in poor product selectivity. Electrochemical depolymerization, particularly direct oxidation, often cleaves bonds non-selectively and produces a complex and varied mixture of LMW aromatic products. Unfinished products are difficult and expensive to separate, which makes both economic recovery and single-targeting of chemicals a major barrier. Furthermore, the process could also result in overoxidation products. The desired LMW monomeric phenols are often more reactive and easier to oxidize than the original lignin polymer. Consequently, as soon as these valuable products are formed, they are attacked either by the electrode or by oxidizing mediators and degraded into low-value carboxylic acids or they are completely mineralized to CO_2_. This severely diminishes the maximum achievable product yield. The last important challenge is that the field is actually constrained by limited electrolyte options. Alkaline aqueous solutions are the most common, but these exacerbate the competing water-splitting reaction. While ionic liquids (ILs) offer excellent lignin solubility and high electrochemical stability, their high cost and potential toxicity are significant drawbacks for a bulk-scale industrial process.

The challenges mentioned above directly highlight the opportunities for innovation in the field. The most critical area for future research is the development of advanced electrocatalysts. The goal is to design highly selective and stable materials (for both anodes and cathodes) that could preferentially catalyze lignin bond cleavage at low overpotentials while simultaneously suppressing the competing water electrolysis reaction. To overcome overoxidation products, the design and interpretation of in situ product-removal techniques offers a valuable opportunity. Continuously separating LMW products from a system as they are formed protects them from degradation. Membrane ultrafiltration appears to be one of the most promising approaches for scalable and continuous operation. There also is a significant need for new, cost-effective, and green electrolytes. Solvents that could efficiently dissolve lignin, possess high ionic conductivity, and exhibit a wide electrochemical stability window are essential. Deep eutectic solvents (DES) represent one such promising avenue of exploration. Also, the integration of hybrid systems, such as combining highly selective microbial or enzymatic catalysis with electrochemical regeneration, offers a promising path forward. These systems, along with photoelectrochemical approaches that directly utilize sunlight, could dramatically improve both the selectivity and sustainability of the process. Realistically, a commercially viable and scaled-up electrochemical lignin conversion process remains a long-term goal. Achieving it will require more fundamental study to understand the complex reaction mechanisms at the electrode–electrolyte–lignin interface, which in turn will enable the rational design of more efficient catalysts, solvents, and reactor systems.

Electrochemical lignin valorization offers a highly promising and sustainable pathway for biomass utilization; however, critical distinctions exist among the various available strategies. Based on the current state of the art, direct anodic oxidation in alkaline media stands as the most technologically mature route, primarily due to its extensive exploration for producing value-added aldehydes like vanillin and syringaldehyde. Conversely, regarding product selectivity, electrocatalytic hydrogenation (ECH) employing non-noble transition metal cathodes (such as Cu) represents the most selective route, because it allows for the targeted cleavage of β-O-4 ether linkages while preventing over-reduction in the aromatic rings. The choice of an electrochemical route must be strictly tailored to the targeted end-products. Oxidative pathways and mild ECH show the most promise for generating highly functionalized aromatic monomers for the fine chemical industry. By contrast, deep ECH, often coupled with hydrodeoxygenation on noble metal catalysts, is the most viable trajectory for producing completely saturated cycloalkanes and drop-in liquid fuels. Despite these advancements, several critical technical bottlenecks remain as serious barriers to industrial implementation. Chief among these is rapid anode deactivation caused by the repolymerization of phenolic intermediates, which drastically reduces current efficiency. Furthermore, characteristics such as inherent recalcitrance and poor solubility of raw lignin in standard aqueous electrolytes continue to limit mass transfer. Ultimately, solving the persistent issue of product overoxidation through the development of continuous in situ product-removal systems such as either membrane reactors or liquid–liquid extraction will be the most decisive factor in transitioning these electrochemical strategies from the laboratory bench to commercial-scale biorefineries.

## Data Availability

No new data were created or analyzed in this study. Data sharing is not applicable to this article.
